# Pooling for SARS-CoV-2 control in care institutions

**DOI:** 10.1186/s12879-020-05446-0

**Published:** 2020-10-12

**Authors:** Jorge Julio Cabrera Alvargonzalez, Sonia Rey Cao, Sonia Pérez Castro, Lucía Martinez Lamas, Olaia Cores Calvo, Julio Torres Piñon, Jacobo Porteiro Fresco, Julio Garcia Comesaña, Benito Regueiro Garcia

**Affiliations:** 1 Microbiology and Infectology Research Group, Galicia Sur Health Research Institute (IIS Galicia Sur), SERGAS-UVIGO, Vigo, Spain; 2 Microbiology Department, Complexo Hospitalario Universitario de Vigo (CHUVI), Sergas, Vigo, Spain; 3 Universidade de Vigo, Vigo, Spain; 4 CINTECX, Universidade de Vigo, GTE, Vigo, Spain; 5 Management Department, Complexo Hospitalario Universitario de Vigo (CHUVI), Sergas, Vigo, Spain; 6 Microbiology and Parasitology Department. Medicine and Odontology, Universidade de Santiago, Santiago de Compostela, Spain

**Keywords:** SARS-CoV-2, Pooling, Detection, Care homes, Low prevalence, Transmission control

## Abstract

**Background:**

Workers and residents in Care Homes are considered at special risk for the acquisition of SARS-CoV-2 infection, due to the infectivity and high mortality rate in the case of residents, compared to other containment areas. The role of presymptomatic people in transmission has been shown to be important and the early detection of these people is critical for the control of new outbreaks. Pooling strategies have proven to preserve SARS-CoV-2 testing resources.

The aims of the present study, based in our local experience, were (a) to describe SARS-CoV-2 prevalence in institutionalized people in Galicia (Spain) during the Coronavirus pandemic and (b) to evaluate the expected performance of a pooling strategy using RT-PCR for the next rounds of screening of institutionalized people.

**Methods:**

**A total of** 25,386 Nasopharyngeal swab samples from the total of the residents and workers at Care Homes in Galicia (March to May 2020) were individually tested using RT-PCR. Prevalence and quantification cycle (Cq) value distribution of positives was calculated. Besides, 26 pools of 20 samples and 14 pools of 5 samples were tested using RT-PCR as well (1 positive/pool). Pooling proof of concept was performed in two populations with 1.7 and 2% prevalence.

**Results:**

Distribution of SARS-CoV-2 infection at Care Homes was uneven (0–60%). As the virus circulation global rate was low in our area (3.32%), the number of people at risk of acquiring the infection continues to be very high. In this work, we have successfully demonstrated that pooling of different groups of samples at low prevalence clusters, can be done with a small average delay on Cq values (5 and 2.85 cycles for pools of 20 and 5 samples, respectively).

**Conclusions:**

A new screening system with guaranteed protection is required for small clusters, previously covered with individual testing. Our proposal for Care Homes, once prevalence zero is achieved, would include successive rounds of testing using a pooling solution for transmission control preserving testing resources. Scale-up of this method may be of utility to confront larger clusters to avoid the viral circulation and keeping them operative.

## Background

Severe acute respiratory syndrome coronavirus 2 (SARS-CoV-2) has caused more than 454,000 deaths since late 2019 [1]. Screening of Care Homes has been critical to limit the mortality rate in Galicia (Spain). Direct viral detection by real time RT-PCR was useful to identify people with potential SARS-CoV-2 transmission risk. Limited stocks and restrictions in test capacity prevented a higher number of RT-PCR tests per day.

Pooling strategies have proven to preserve SARS-CoV-2 testing resources and time with an increase in testing capability of the 69% for an incidence rate of SARS-CoV-2 infection of 10% or less [2–8], but it could be associated with a decrease in detection [9, 10]. Main limitations could be the preanalytical step, the sample viral load or the increase of the limit of detection of the individual sample [6].

The rationale in this study is to develop a new strategy based on initial individual identification of positive coronavirus cases in order to organize low prevalence clusters, followed by a serial pooling strategy testing of these clusters, in order to control areas free of virus circulation, allowing them to be fully operative.

## Methods

### Samples

Nasopharyngeal swab samples were obtained from residents and workers at Care Homes in Galicia (March to May 2020) and conserved in viral transport medium. The study protocol (2020/298) was approved by the Galician network of committees of research ethics.

### Care homes screening

Samples were mixed 1:1 with cobas® omni lysis reagent (43% guanidine thiocyanate) for viral inactivation before individual testing. The Open Reading Frame (ORF) 1/b non-structural region of SARS-CoV-2 and the envelope E-gene of Sarbecovirus were detected with the cobas® SARS-CoV-2 test (Roche Diagnostics, NJ, USA) on the cobas® 6800 system (Roche Diagnostics). For all RT-PCRs in this study, a sample was considered positive if at least one target was detected (quantifying cycle -Cq- below 40).

### Pooling testing

Pooling of samples was performed by the QIAgility instrument (QIAgen) using 50–150 μL of each sample.

For positivity assessment, selected positive samples were processed individually and by pooling (1 positive/pool) using the MagCore® HF16 Plus system (RBC Bioscience) and the Allplex™2019-nCoV assay (Seegene In, Seoul, South Korea) on the CFX-96 system (Bio-Rad Laboratories, Hercules, CA, USA). Positive samples detected during Care Homes screening with Cq value below the third quartile were selected. For a proof of concept, screening of selected Care Homes was performed using a pooling strategy by the STARlet instrument (Microlab) with STARMag 96 × 4 Universal Cartridge Kit for automated extraction and PCR set-up. The RNA-dependent RNA polymerase (RdRP) and nucleocapsid (N) genes of SARS-CoV-2 and the E gene were detected. Selection was performed by prevalence observed during the screening step.

### Statistical analysis

**Global SARS-CoV-2 prevalence and 95% confidence interval were calculated.** Distribution of care institutions by SARS-CoV-2 prevalence and a summary of Cq values of positive samples were calculated.

Differences in Cq values (mean and range) obtained by individual and pooling testing strategies were calculated for each target. The Cq values were considered as 41 in case of undetectable result.

Global sensitivity and reduced number of tests were calculated for screening with pooling. R version 3.5.1 http://www.R-project.org/

## Results

### SARS-CoV-2 screening of care homes

**Fig. 1:**
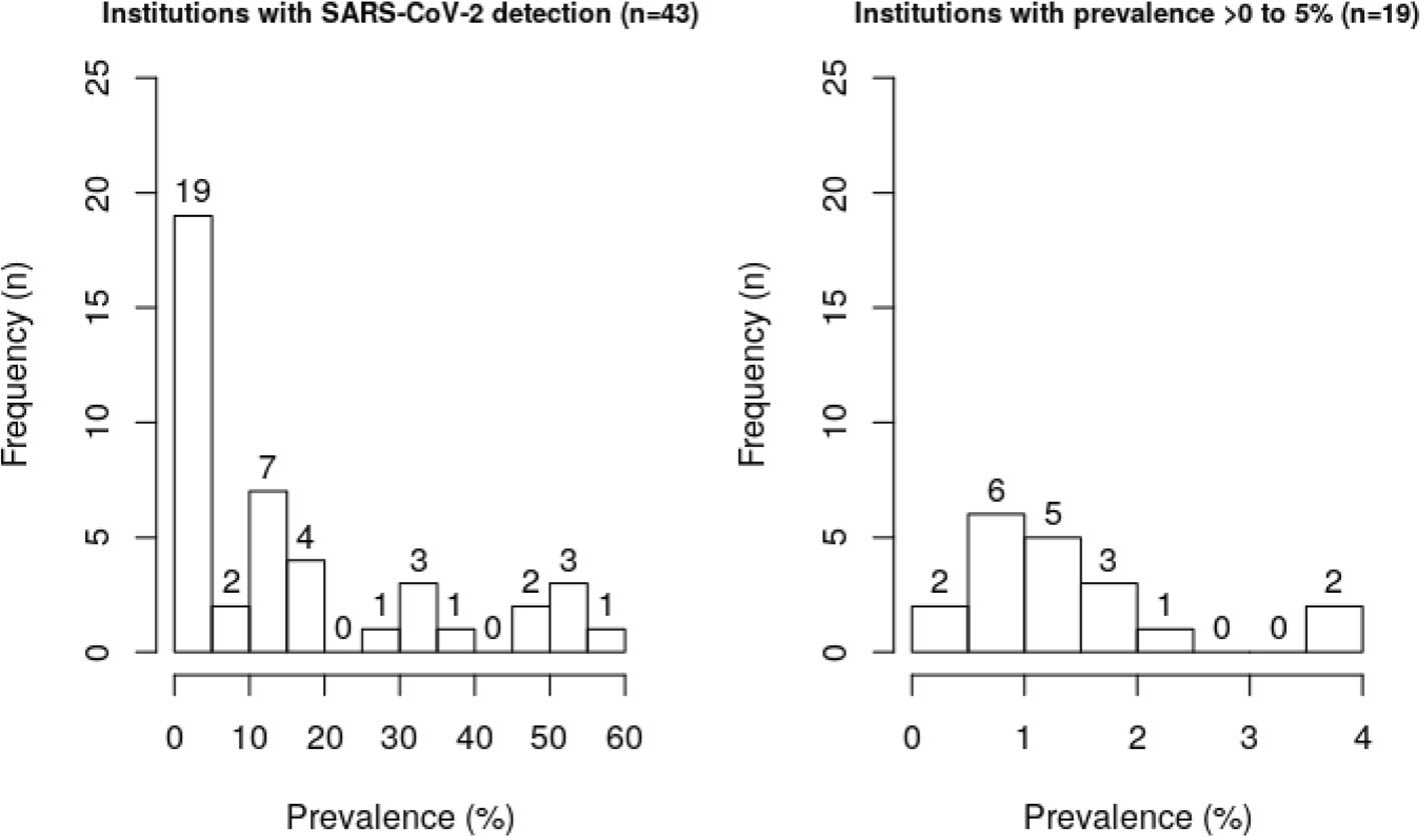
Distribution of Care Institutions by SARS-CoV-2 prevalence, excluding those with prevalence zero

During the Coronavirus pandemic, SARS-CoV-2 prevalence was obtained by individually testing of 25,386 people from 306 Galician Care Homes: 16,477 residents, 8599 workers and 310 not specified. The mean age of workers and residents was 44.25 years (min 18, max 69) and 80.07 years (min 3, max 109), respectively. SARS-CoV-2 was detected in 852 people (3.32, 95% CI: 3.10–3.54%). The distribution of institutions by SARS-CoV-2 prevalence is shown in Fig. 1. A total of 282 institutions (21,861 people) had SARS-CoV-2 prevalence < 4%, including 263 institutions (19,091 people) with prevalence zero. Prevalence from 5 to 10% was observed in 2 institutions (389 people), from 10 to 20% in 11 institutions (1817 people) and from 20 to 60% in 11 institutions (1309 people).

Cq value distribution for positive samples was as follows: minimum 15.03/15.41, 1st quartile 21.86/22.55, median 26.41/27.54, 3rd quartile 31.36/33.60 and maximum 35.86/39.06 for ORF1b and E gene, respectively. Additional data of distribution and Cq values of positive samples detected during SARS-CoV-2 screening of Care Homes are available as additional files 1, 2 and 3.

### Pool positivity assessment

The selection of the optimal pool size should be made before the implementation of pooling testing. With non-overlapping pools, only positive pools will be retested. The reduction of the expected number of tests depends on the prevalence, the initial pool size and the number of stages for the pooling algorithm. In fact, it is generally accepted that 5% could be the prevalence threshold to achieve a 50% reduction in the expected number of tests per individual. On the other hand, the sensitivity and specificity of the global process depends on the analytical characteristics of the test and on the number of times one sample is retested. Differences in the expected number of tests per individual, based on mathematical simulations, could help to choose the best set of pool sizes. According to other authors [11], for prevalence between 1 and 2%, sensitivity 95% and specificity 100%, the optimal pool size would be between 25 and 16 samples and the optimal sub pool size would be between 4 and 5 samples. In order to minimize the false negative factor for pooled testing recently defined [3] and to standardize the pooling method, pools of twenty samples (P20) and sub pools of five samples (SP5) were selected.

**Fig. 2:**
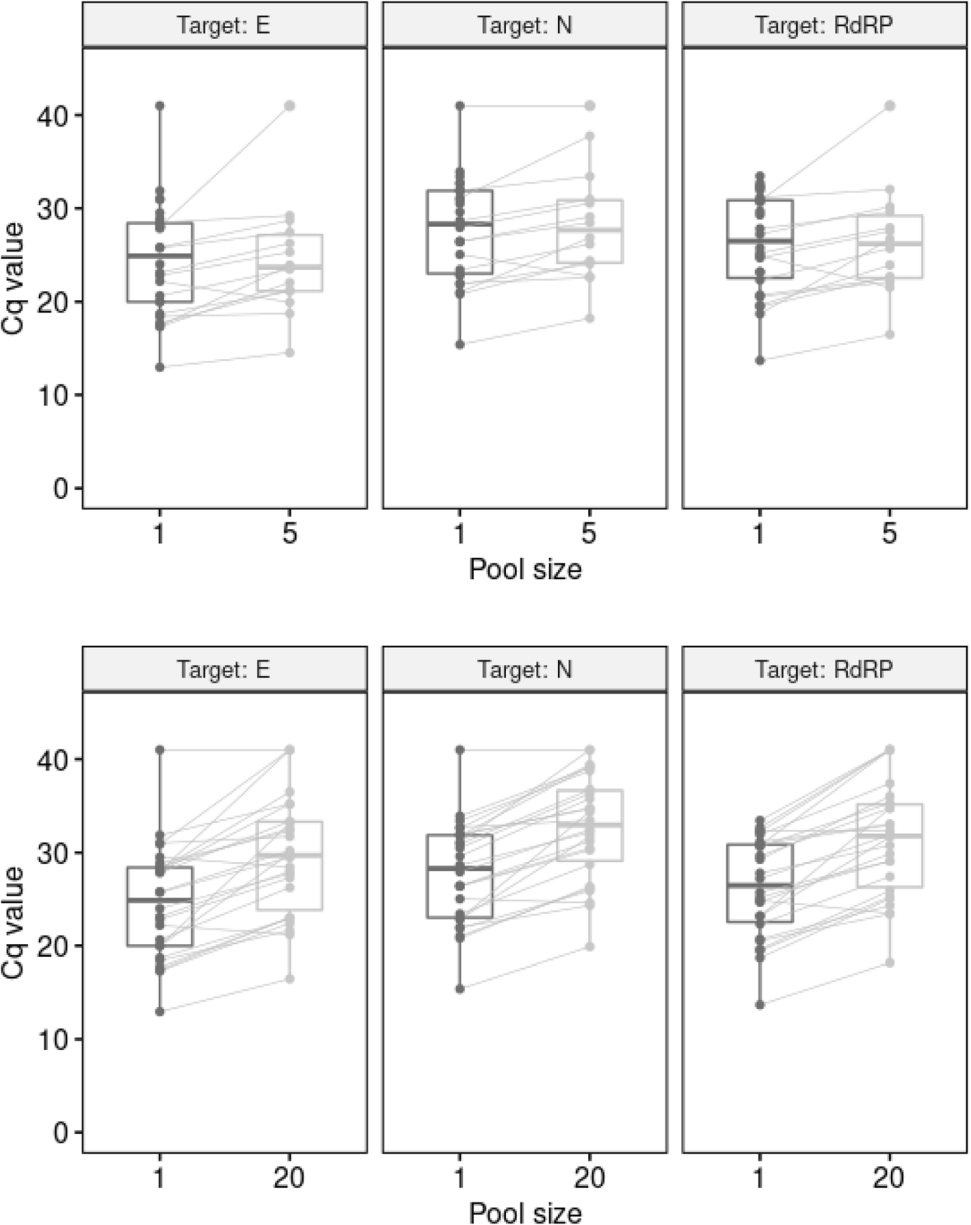
Boxplot of paired Cq values obtained for pooling and individual testing. Values are shown for each gene and pool size

Test performance of twenty-six P20 and fourteen SP5 was studied. Each pool included one positive sample. A total of twenty-six positive samples were tested. Mean Cq values were 27.43 and 28.68 for ORF1/b and E gene, respectively. A boxplot of paired Cq values is shown in Fig. 2. All positive samples yielded a global positive result when tested in P20 or SP5. Sensitivity of E, RdRP and N gene was, respectively, 88.5% (23/26), 84.6% (22/26) and 96.1% (25/26) for P20. Sensitivity was 92.9% (13/14) for the three targets for SP5.

Mean delay in the Cq values (Cq pool– Cq positive sample) was 5.02 cycles for the P20 and 2.85 cycles for the SP5 (Table 1). An example of the amplification curves obtained for one particular sample is shown in additional files 4, 5 and 6. The N gene was not detected by Allplex™2019-nCoV assay in one specific sample independently of pooling or individual testing.

**Table 1:** Differences in quantification cycle (Cq) values between pooling and individual testing by Allplex™2019-nCoV assay

Pool size	Parameter	Testing Condition	E gene Mean (range)	N gene Mean (range)	RdRP gene Mean (range)
P20 (*n* = 26)	Cq value	individual	24.63 (12.96, > 40)	27.79 (15.4, > 40)	26.23 (13.69, 33.45)
		pool	29.67 (16.48, > 40)	32.65 (19.9, > 40)	31.39 (18.2, > 40)
		difference	5.04 (−1.06, 13.15)	4.86 (−0.42, 11.52)	5.16 (−1.39, 11.57)
SP5 (*n* = 14)	Cq value	individual	21.38 (12.96, 28.48)	25.92 (15.4, > 40)	23.34 (13.69, 31.15)
		pool	24.49 (14.5, > 40)	28.30 (18.23, > 40)	26.42 (16.48, > 40)
		difference	3.11 (−2.26, 13.01)	2.37 (−2.32, 6.66)	3.08 (−3.33, 10.22)

*P20* Pool of 20 samples, *SP5* sub pool of 5 samples, *Cq* Quantification cycle

### Proof of concept

Samples from Care Homes selected by prevalence were retrospectively tested in pools using the following algorithm: P20, SP5 when positive, individual analysis when positive. A first simulation was performed with 100 samples from 2% (95% CI: 0.24–7.04%, 2/100) prevalence Care Homes. Five P20 were tested. As 2 positive pools were obtained, 8 SP5 were processed. Two SP5 were positive, so 10 samples were tested individually. Two samples were positive. Number of tests was reduced 77% (0.23 tests per individual).

A second simulation included 60 samples from 1.7% (95% CI: 0.04–8.94%, 1/60) prevalence institutions. Three P20, 4 SP5 and 5 individual samples were tested. One sample was positive. Number of tests was reduced by 80% (0.20 tests per individual).

## Discussion

A global SARS-CoV-2 seroprevalence of 5% in Spain [12] and a global viral prevalence around 3% at Care Homes reported in the present study, suggest that the number of people at risk of acquiring the infection continue to be very high. The role of transmission before symptoms has been shown to be important, presymptomatic / asymptomatic individuals may contribute to it [13, 14]. For these reasons their early detection seems critical to prevent further outbreaks. To control the spread of the virus, it is essential to detect as many infected individuals as possible, as quickly as possible to trace down and test possible contacts [15].

We performed the screening of 306 Care Homes (25,386 determinations) in workers and residents using individual testing by RT-PCR. With a prevalence < 2% for more than 85% people in Care Homes, pooling could achieve maximum usefulness. After reviewing the literature, and due to the absence of accumulated experience with this type of strategy for SARS-CoV-2, a more conservative pool size of 20 and sub pool size of 5 samples were chosen [2, 3, 6–8, 16].

Two tests authorized by the Food and Drug Administration were available at our laboratory. Both have shown suitable specificity and sensitivity for clinical diagnosis, but specific studies will be required for assessing their performance in pooling conditions. The choice of the Allplex™2019-nCoV Assay was due to the flexibility and adaptability in the automation process useful for future interventions. Additionally, although it has been established a moderate mutation rate of SARS-CoV-2 [17–19], the possibility of detecting three targets could increase the possibilities of detection [18, 20].

As previous studies [9, 10, 16], our results using pools showed an increase of 3–5 cycles in the Cq value between pooled tests and individual positive samples. The pooling strategy was associated with a decreased sensitivity for individual targets (4–7%). Nevertheless, it has not carried out loss of global sensitivity in pools for samples included in this study. Samples of this study have been selected in order to represent those with Cq value within the first three quartiles observed in our population. A 100% global sensitivity was also achieved when testing Care Homes with prevalence around 2%, reducing until 80% the number of tests.

### Proposed methodology

Here there is our proposal for introducing the pooling strategy for screening purposes in Care Institutions: When an institution with prevalence zero is characterized, successive rounds of pooling testing would be the option for transmission control. The maximum interval between rounds would be adjusted to avoid the loss of detection of infected people who could be in a phase of low viral load. The incubation period has been reported to be highly variable with an estimated average of 5–6 days [13, 21–24]

Limitations of this study were the limited number of samples included. Testing more negative samples would allow us to assess specificity and the risk of contamination along the processing. There is a likelihood of obtaining false negative results when a pooling strategy is introduced. Mainly, low quality samples cannot be discarded from the pools and the dilution could reduce the ARN concentration below the limit of detection. In this study we have focused on demonstrating that any pool containing until 20 individual samples from highly infectious people would be detected.

This work has shown the prevalence of SARS-CoV-2 in Spanish Care Homes during the Coronavirus pandemic. Prevalence differences shown between Institutions should address the interventions for viral transmission control. Few studies have assessed the performance of pooling for SARS-CoV-2 detection by rRT-PCR in real conditions, especially when aiming to keep areas free of virus circulation to be operative and functional.

## Conclusions

Sample pooling could be a new testing strategy relevant for maintaining low level or no transmission among institutionalized people. Our proposal for Care Homes, once prevalence zero is achieved, would include successive rounds of testing using a pooling solution for transmission control preserving testing resources. Scale-up of this method may be of utility to confront larger clusters to avoid the viral circulation and keeping them operative. Further studies with self-sampling methods, modular systems and more specific pooling strategies will be necessary for the process improvement.

## Supplementary information

**Additional file 1.** SARS CoV-2 prevalence. Global prevalence is shown on the left. Stacked bar charts show Care Home prevalence obtained by individual testing for Care Homes with SARS-CoV-2 infections and without infection (prevalence zero).

**Additional file 2.** Distribution of SARS-CoV-2 RT-PCR Cq value. Summary of the distribution of Cq values of Care Homes with more than 5 positives. It is shown for each detected target. Samples were tested individually.

**Additional file 3.** Age Distribution. Distribution of age of Care Home residents and workers individually tested.

**Additional file 4.** E gene amplification curves. Example of amplification curves (E gene) obtained for the same sample processed individually and in pools of 5 (P5) and 20 (P20) samples. Obtained Cq values were 26.20, 29.31 and 30.82 for the individual sample, P5 and P20, respectively.

**Additional file 5.** N gene amplification curves. Example of amplification curves (N gene) obtained for the same sample processed individually and in pools of 5 (P5) and 20 (P20) samples. Obtained Cq values were 28.69, 31.99 and 33.26 for the individual sample, P5 and P20, respectively.

**Additional file 6.** RdRP gene amplification curves. Example of amplification curves (RdRP gene) obtained for the same sample processed individually and in pools of 5 (P5) and 20 (P20) samples. Obtained Cq values were 28.80, 31.49, 31.79 for the individual sample, P5 and P20, respectively.

## Data Availability

The datasets used and/or analysed during the current study are available from the corresponding author on reasonable request. All data and materials were obtained working under Servizo Galego de Saúde, Consellería de Sanidade of Xunta de Galicia (institutions belonging to our National Health Public System) and are under their regulations.

## Abbreviations

SARS-CoV-2: Severe acute respiratory syndrome coronavirus-2
RT-PCR: Reverse transcription polymerase chain reaction
Cq: quantification cycle
ORF: Open reading frame
P20: Pool of twenty samples
SP5: Subpool of five samples
