# Supplementary material for: Pooling for SARS-CoV-2 control in care institutions
Source: BMC Infect Dis. 2020 Oct 12;20:745. doi: 10.1186/s12879-020-05446-0 (PMC7549089; doi:10.1186/s12879-020-05446-0)
Supplement: Supplementary file 1 — Additional file 1. SARS CoV-2 prevalence. Global prevalence is shown on the left. Stacked bar charts show Care Home prevalence obtained by individual testing for Care Homes with SARS-CoV-2 infections and without infection (prevalence zero). [file 12879_2020_5446_MOESM1_ESM.html]

 


010020030040050005010015020025030096.7%3.32%NegativePositiveSARS-CoV-2 PREVALENCENumber of individualsNumber of individualsCare HomeCare Homes with non-zero prevalenceCare Homes with zero prevalence

plotly-logomark
