# Supplementary material for: Pooling for SARS-CoV-2 control in care institutions
Source: BMC Infect Dis. 2020 Oct 12;20:745. doi: 10.1186/s12879-020-05446-0 (PMC7549089; doi:10.1186/s12879-020-05446-0)
Supplement: Supplementary file 2 — Additional file 2. Distribution of SARS-CoV-2 RT-PCR Cq value. Summary of the distribution of Cq values of Care Homes with more than 5 positives. It is shown for each detected target. Samples were tested individually. [file 12879_2020_5446_MOESM2_ESM.html]

 


152025303540ORF1bGene EDISTRIBUTION OF SARS-CoV-2 RT-PCR Cq VALUECare HomesCq value

plotly-logomark

ORF1b(CH\_243, median: 29.70)(CH\_243, q1: 25.61)(CH\_243, q3: 31.27)(CH\_243, min: 18.46)(CH\_243, max: 34.29)(CH\_243, mean ± σ: 28.42 ± 4.48)
