# Supplementary material for: Pooling for SARS-CoV-2 control in care institutions
Source: BMC Infect Dis. 2020 Oct 12;20:745. doi: 10.1186/s12879-020-05446-0 (PMC7549089; doi:10.1186/s12879-020-05446-0)
Supplement: Supplementary file 3 — Additional file 3. Age Distribution. Distribution of age of Care Home residents and workers individually tested. [file 12879_2020_5446_MOESM3_ESM.html]

 


ResidentWorker020406080100ResidentWorkerAGE DISTRIBUTIONAge (years)

plotly-logomark
