# Supplementary figures and images for: Pooling for SARS-CoV-2 control in care institutions
Source: BMC Infect Dis. 2020 Oct 12;20:745. doi: 10.1186/s12879-020-05446-0 (PMC7549089; doi:10.1186/s12879-020-05446-0)

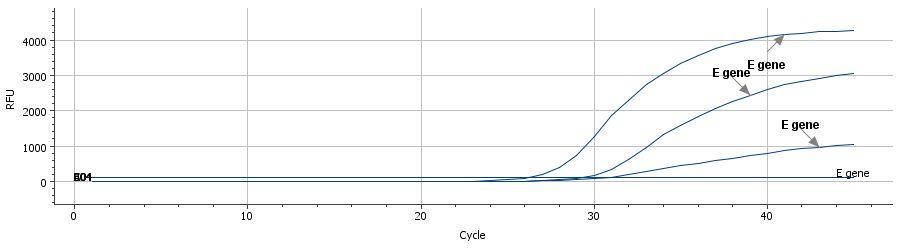

Supplement: Supplementary file 4 — Additional file 4. E gene amplification curves. Example of amplification curves (E gene) obtained for the same sample processed individually and in pools of 5 (P5) and 20 (P20) samples. Obtained Cq values were 26.20, 29.31 and 30.82 for the individual sample, P5 and P20, respectively. [file 12879_2020_5446_MOESM4_ESM.png]

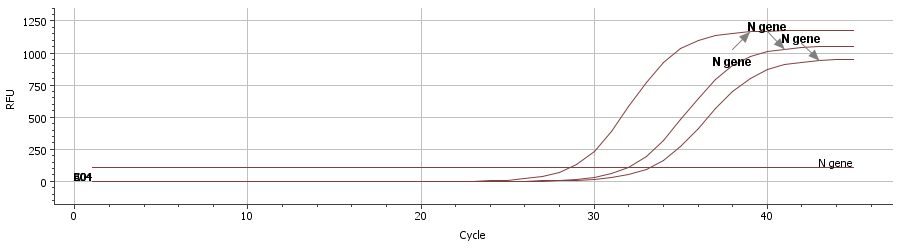

Supplement: Supplementary file 5 — Additional file 5. N gene amplification curves. Example of amplification curves (N gene) obtained for the same sample processed individually and in pools of 5 (P5) and 20 (P20) samples. Obtained Cq values were 28.69, 31.99 and 33.26 for the individual sample, P5 and P20, respectively. [file 12879_2020_5446_MOESM5_ESM.png]

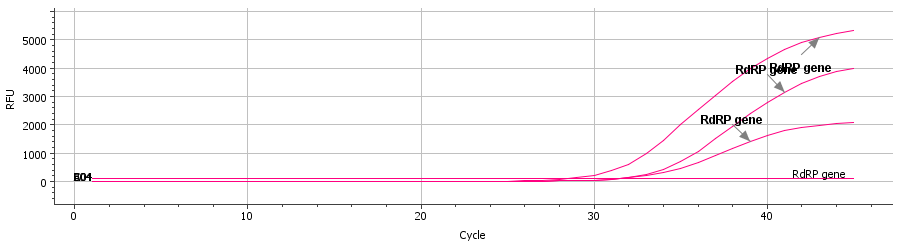

Supplement: Supplementary file 6 — Additional file 6. RdRP gene amplification curves. Example of amplification curves (RdRP gene) obtained for the same sample processed individually and in pools of 5 (P5) and 20 (P20) samples. Obtained Cq values were 28.80, 31.49, 31.79 for the individual sample, P5 and P20, respectively. [file 12879_2020_5446_MOESM6_ESM.png]
